# Flavonoids of Kudzu Root Fermented by *Eurtotium cristatum* Protected Rat Pheochromocytoma Line 12 (PC12) Cells against H_2_O_2_-Induced Apoptosis

**DOI:** 10.3390/ijms18122754

**Published:** 2017-12-19

**Authors:** Bo Zhang, Wen Li, Mingsheng Dong

**Affiliations:** 1 College of Food Science and Technology, Nanjing Agricultural University, Nanjing 210095, China; vip_zhangbo87@163.com; 2 College of Food Science and Technology, Bohai University, Jinzhou 121013, China; 3 Jiangsu Key Construction Laboratory of Food Resource Development and Quality Safe, Xuzhou Institute Technology, Xuzhou 221008, China; wenlisony@126.com

**Keywords:** kudzu root (*Pueraria lobata*), PC12 cell, H_2_O_2_-induced damage, reactive oxygen species, apoptosis

## Abstract

Novel bioactive components have greatly attracted attention as they demonstrate health benefits. Reversed-phase high performance liquid chromatography (RP-HPLC) showed that isoflavonoid compounds of kudzu root (*Pueraria lobata*) fermented by *Eurtotium cristatum* and extracted using de-ionized water were higher active compared with non-fermented. A model of H_2_O_2_-inducd cell damage was built using rat pheochromocytoma line 12 (PC12) cell to observe the protective effect of non-fermented kudzu root (*Pueraria lobata*) (NFK) and fermented kudzu root (*Pueraria lobata*) (FK). Cell viability and apoptosis were analyzed through inverted microscopy and flow cytometry. The level of lactate dehydrogenase, catalase activity, superoxide dismutase, glutathione, and reactive oxygen species (ROS) were evaluated. Results showed that NFK and FK could significantly protect PC12 cell against damage caused by H_2_O_2_-induced oxidative stress. The intracellular antioxidant system was increased, protected the cell membrane inhibit H_2_O_2_-induced apoptosis by scavenging of ROS. Moreover, NFK and FK regulated the cell cycle to prevent cell apoptosis. Isoflavonoid from the kudzu root especially fermented kudzu root with *E. cristatum* are potentially therapeutic drugs against diseases induced by oxidative damage.

## 1. Introduction

Health foods and their by-products have recently attracted considerable attention. Various phytochemical components have been recognized as natural antioxidants, which are used to counteract reactive oxygen species. Among these phytochemical compounds, phenols have demonstrated potent antioxidant activity, as indicated by the reduction of chronic diseases [1]. Consequently, bioactive phenolic acid compounds have received great considerable attention in the food and clinical sectors because of their potential as antioxidants [2]. The beneficial effects of isoflavonoids are attributed to their anti-oxidative and phytoestrogenic properties [3]. Phenolic compounds and their anti-oxidant activity in legume seeds have been reported by several studies [4,5]. Pathogenesis and progression of various human diseases, including cancer, neurodegenerative diseases, and Alzheimer’s disease (AD), are caused by two critical factors, namely, free radical and oxidative stress [6,7]. These diseases are possibly associated with overproduction of reactive oxygen species (ROS) or reactive nitrogen species [8,9].

Fan et al. [10] reported that apoptosis of neuronal cell contributes to the apoptotic pathway, and such contribution is caused by mitochondrial homeostasis, which is affected by major reactive oxygen species (ROS) (e.g., superoxide radical and hydroxyl radical, by energy production, and mediate mitochondrial-dependent pathway). Cells can protect themselves from damages, injury, and apoptosis induced by oxidative stress through their internal antioxidant defense mechanisms [10]. Meanwhile, estrogen therapy is one of the most compelling potential strategies to prevent AD [11]. Dietary flavonoids have received great attention as potential anticancer agents, cardioprotectants, and inhibitors of neuro de-generation because of their antioxidant properties and their ability to modulate signaling pathways [12,13].

Rat pheochromocytoma line 12 (PC12) cell provides a useful model system for neurological and neurochemical studies [14]. In PC12 cell, neuronal apoptosis may be due to different apoptotic pathways: intrinsic or extrinsic for example [15]. H_2_O_2_ is the main component of reactive oxygen species, and it is also an inducer of oxidative stress. Exogenous hydrogen peroxide can induce a variety of cells to cause injury or even apoptosis due to oxidation. In PC12 cells, H_2_O_2_ peroxide can induce intracellular reactive oxygen species (ROS), thereby motivating cell oxidative damage, resulting in the expression of related genes or related apoptotic proteases, resulting in cell apoptosis. [16]. H_2_O_2_ in PC12 cell is recently demonstrated to induce cytotoxicity, as well as membrane and antioxidant enzyme activities, such as superoxide dismutase (SOD) and catalase (CAT) activities, moreover, H_2_O_2_ increases ROS level and caspase-3 activity [17,18].

Lin et al. [19] reported that herbal remedies are a type of alternative medicine that employs traditional herbal treatments to heal illnesses and diseases. Although herbal medicines, which are usually acquired from diverse natural resources, are traditional methods employed outside of conventional medicine, many people worldwide still rely on herbal medicines for health care [19].

Food-derived antioxidants, such as phytochemicals, have recently received increasing attention because of their function as chemopreventive agents against oxidative damages. Lu et al. [14] reported that natural flavonoids displaying a lipophilic chemical structure and antioxidant properties are promising candidates for neurodegenerative intervention. Many plants and cereals were proven have against harmful-free radicals due to the presence of antioxidant compounds such as isoflavones and phenolic acids. Moreover, Zhang et al. [20] have found that puerarin protects PC12 cells against β-amyloid-induced cell injury. Puerarin is one of the major isoflavonoid compound isolated from the root of wild leguminous creepers [21]. Kudzu root (*Pueraria lobata*) protects neurons from oxidative stress-induced apoptosis. However, the effect of kudzu root (*Pueraria lobata*) and fungus-fermented kudzu root on apoptosis of PC12 cell during H_2_O_2_-induced damage has not yet been investigated. This study examined whether *E. cristatum* fermented kudzu root (*Pueraria lobata*) exert neuro-protective effects against H_2_O_2_-induced ROS production and whether fermented kudzu root inhibits apoptosis of PC12 cell.

## 2. Results and Discussion

### 2.1. Special Isoflavonoid Contents of NFK and FK

**Figure 1:**
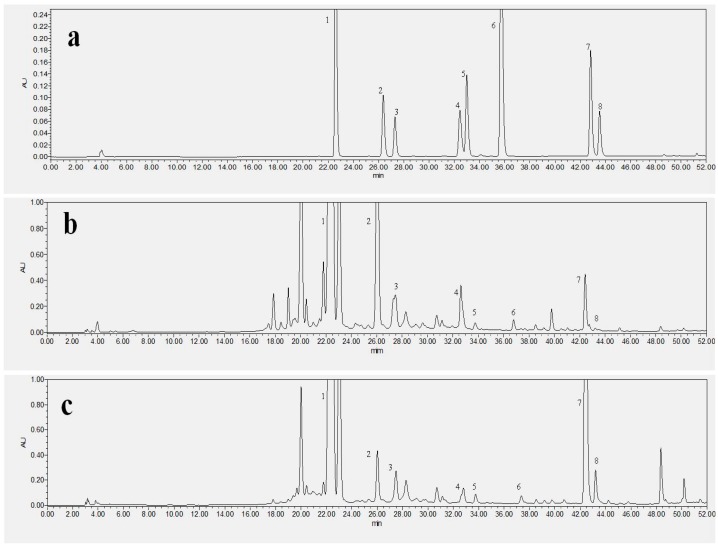
Reversed-phase high performance liquid chromatography (RP-HPLC) chromatogram analyzed the isoflavones of seven standard, non-fermented kudzu root (NFK), fermented kudzu root (FK). (**a**) including (1) Puerarin; (2) Daidzin; (3) Glycitin; (4) Genistin; (5) Ferulic acid; (6) Daidzein; (7) Glycitein; (8) Genistein; (**b**) NFK; (**c**) FK.

Figure 1 and Table 1 showed the NFK and FK samples contain high amount isoflavonoid. Wang et al. [22] reported that puerarin, daidzein, and genistein are the major isoflavonoids in kudzu root (*Pueraria lobata*) [23]. Table 1 showed that seven isoflavonoid compounds and one phenolic acid, namely puerarin, daidzin, glycitin, genistin, daidzein, glycitein, genistein and shikimic acid have changed during fermentation time as revealed by RP-HPLC. *E. cristatum* could increase or decrease the isoflavonoid and phenolic acid contents of kudzu root, for instance, daidzein level was higher in FK (188.77 ± 12.47 µg/g. Dry Weight) than in NFK (145.62 ± 23.91 µg/g. DW) extracted using deionized water. Additionally, the puerarin contents of FK were 2395.26 ± 78.65 µg/g. DW, compared with that of NFK (1903.56 ± 62.88 µg/g. DW) extracted using deionized water. *E. cristatum* fermented kudzu root (*Pueraria lobata*) may be produced some enzyme which could be cut off glucoside bond and then increased the isoflavonoid contents [24]. The microbe grew and metabolized on the substrate could be produce some chemical substances contribute to the isoflavonoid and phenolic acid contents [25]. He et al. [26] recently demonstrated that FK and NFK effectively prevent H_2_O_2_-induced apoptosis of PC12 cell. Li et al. [27] also reported that isoflavonoid significantly prevents cells apoptosis and that estrogen may directly act on nerve growth factors and increase the number of nigral dopaminergic neurons. Some studies suggest that tea polyphenols act as antioxidants by scavenging free radicals and chelating metals, whereas other works suggest that ployphenols act as pro-oxidants, increasing the levels of intracellular ROS and contributing to mitochondria-mediated apoptosis [28].

### 2.2. Protection of PC12 Cell from H_2_O_2_-Induced Damage as Revealed by Cell Morphology Analysis through Phase Contrast Microscopy

**Figure 2:**
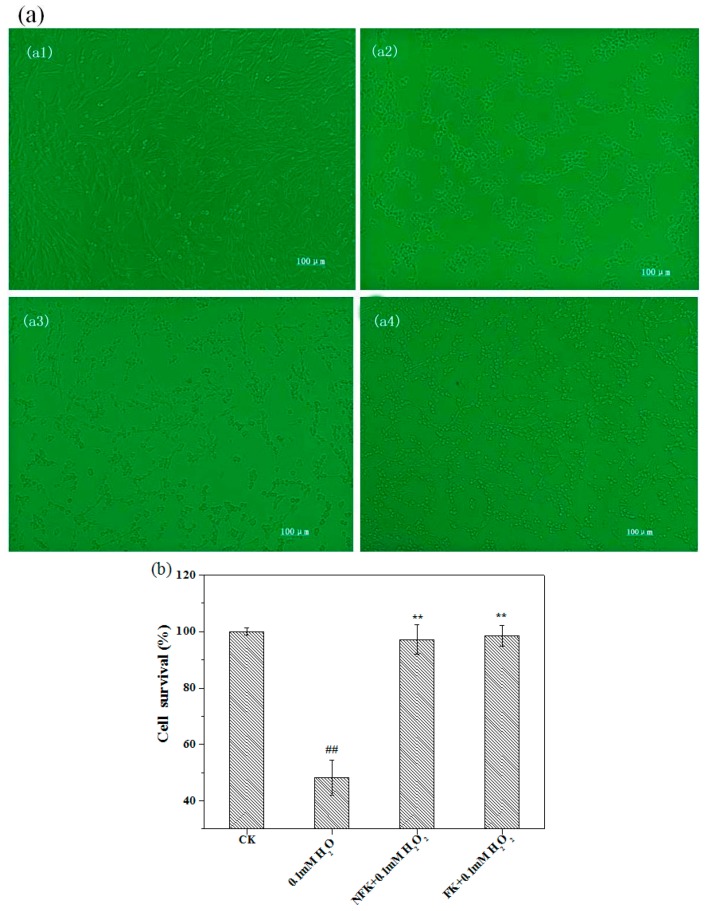
Effects of NFK and FK on PC12 cell damaged by 0.1 mM H_2_O_2_. (**a**) Cell microscope observed under an inverted microscope, (**a1**) CK; (**a2**) damage group; and (**a3**,**a4**) sample group (scale: 100 µm); (**b**) Cytotoxicity of NFK (10 mg/mL) and FK (10 mg/mL) on PC12 cells. Data are presented as mean ± S.D. (*n* = 3). ^##^ *p* < 0.01 indicates significant differ between the control group and damage group, ** *p* < 0.01 indicates significant difference between sample group and damage group.

The morphology of the cells was observed under an inverted microscope, the normal cells grew better than the cells damaged by H_2_O_2_, that is, the normal cells showed clear boundary and were apparently ellipse and full although they were dendritic (Figure 2a(a1)). When the cells were treated with 0.1 mM H_2_O_2_ for 2.5 h, the cells shrunk and appeared round, the spacing among treated cells were significantly larger than normal cells, moreover, the cells were fragment, agminat, and necrotic (Figure 2a(a2)). The cells pre-incubated with NFK and FK for 0.5 h and then exposed to H_2_O_2_ appeared more intact than the cells of the damage group and appeared more similar to the cells of the control group (Figure 2a(a3,a4)).

**Figure 3:**
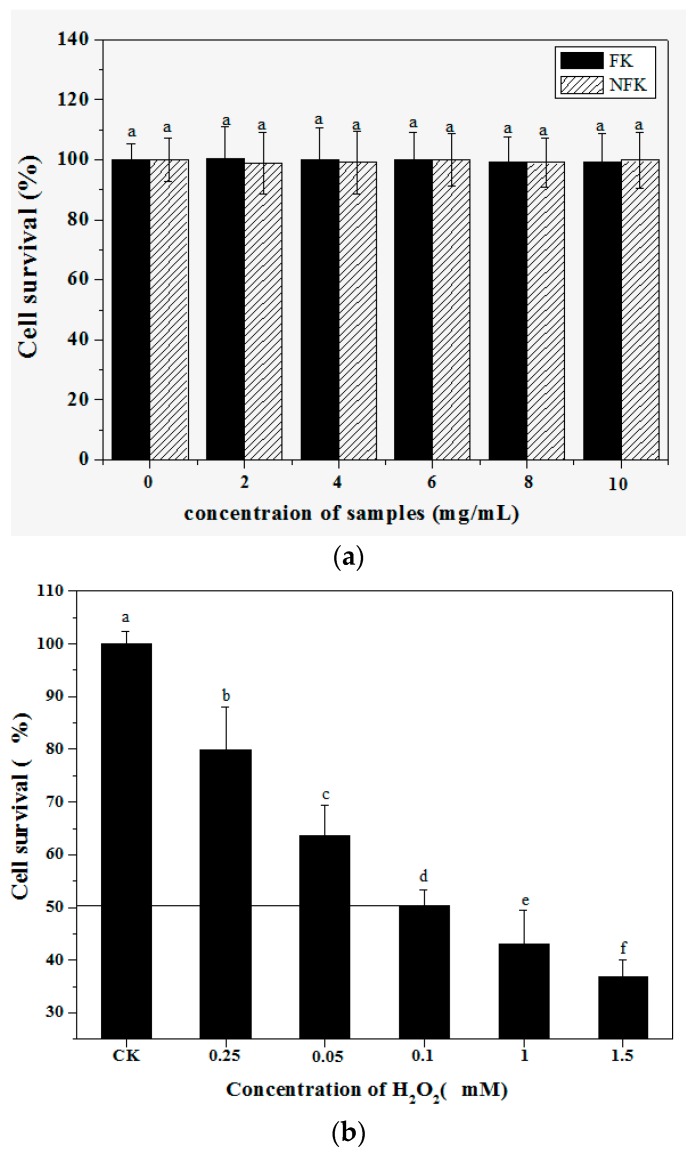
(**a**) The different concentration of NFK and FK on PC12 cells viability. Each value is expressed as the mean ± SD (*n* = 3). In a column, the same superscript letters indicates that the difference between NFK and FK is not significant (*p* > 0.05); (**b**) The oxidant model was evaluated by different concentration of H_2_O_2_ on PC12 cells, which treated for 0.5 h exposed H_2_O_2_. Each value is expressed as the mean ± SD (*n* = 3). Within a column, values with the different superscript letters are significantly different from each other at *p* < 0.05, line indicated the IC_50_ value.

MTT assay was used to investigate the effects of NFK and FK on viability of PC12 cell damage or toxicity, the results expressed in Figure 3a. The IC_50_ of the viability of PC12 cell was disrupted by 0.1 mM H_2_O_2_ within 2.5 h incubation as shown in Figure 3b, and this H_2_O_2_ concentration was used in the damage model. MTT was used to determine the survival rate of cells pre-incubated with FK and NFK for 0.5 h, treated with 0.1 mM H_2_O_2_, and incubated for 2.5 h. Figure 2b showed that effectively protected the PC12 cell exerted by FK extracted using deionized water was 98.52 ± 3.61%. Based on cell survival, the protective effect of FK and NFK could be against H_2_O_2_-induced damage.

### 2.3. LDH, CAT, and SOD Activities, GSH Content, and ROS Levels of PC12 Cell

LDH (Lactate dehydrogenase) is a biological macromolecule that cannot be released from a normal cell unless cell membrane is damaged by an extracellular substance. To investigate the protective effect NFK and FK on cells, the LDH release was detected and the level of cells damage caused by 0.1 mM H_2_O_2_ in pre-incubated samples was determined. Table 2 showed that the LDH release (566.45 ± 79.43 U/L) in PC12 cell in the damage group treated with 0.1 mM H_2_O_2_ for 2.5 h significantly (*p* < 0.01) increased compared with that in the control group. After being pre-protected for 0.5 h with NFK and FK extracted using deionized water, the LDH release was 468.45 ± 19.20 and 443.80 ± 33.26 U/L of the cells, respectively, it were significantly (*p* < 0.01) decreased compared with that in the damage group. After treatment with NFK and FK, the viability of PC12 cell was significantly reversed as indicated by a remarkable improvement in MTT (3-(4,5-Dimethylthiazol-2-yl)-2,5-diphenyltetrazolium bromide) value and a reduced LDH release, indicating that the samples could prevent cell damage and injury after H_2_O_2_ treatment in PC12 cell model [29].

Toxicity caused by H_2_O_2_ is normally accompanied by increased intracellular oxidative stress. Therefore, the effects of NFK and FK on antioxidant were analyzed by determining the different antioxidant systems in PC12 cell (Table 2). SOD being a natural superoxide free radical scavenging factor in the organism, is generally regarded as the main line of defense against tissue and cellular damage caused by cytotoxic reactive oxygen species [30,31]. SOD indirectly indicates the level of intracellular radicals. CAT is an enzyme that can decompose hydrogen peroxide into oxygen and water, wherein the hydrogen acts as metabolite during metabolic processes, moreover, CAT is the important antioxidant enzyme that decomposes hydrogen to reduce damage in cells [32]. GSH is an abundant natural neuronal antioxidant that plays a critical role in cell survival against oxidative stress. Intracellular ROS level induced by H_2_O_2_ in PC12 cells was examined by using the special fluorescent dye DCFH-DA, which enhances fluorescent intensity following generation of reactive intracellular metabolites [33,34].

**Figure 4:**
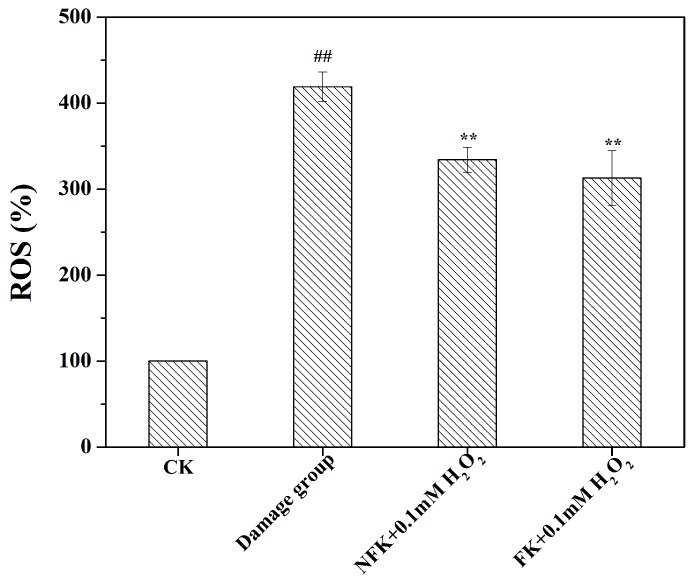
Intracellular ROS level of NFK (10 mg/mL) and FK (10 mg/mL) in PC 12 cells. The fluorescent probe DCFH-DA was used to the cell ROS level. Data are presented as mean ± S.D., (*n* = 3). ^##^ *p* < 0.01 compared with the control cells; ** *p* < 0.01 compared with the damage group.

Table 2 and Figure 4 showed that 0.1 mM H_2_O_2_ significantly reduced the levels of the anti-oxidant defense enzymes CAT and SOD, and GSH content, whereas ROS level increased in H_2_O_2_ treated PC12 cell. By contrast, NFK and FK increased the levels and enhanced the activities of CAT and SOD, and GSH content, whereas they reduced the ROS level in pre-treated PC12 cell. The pretreatment could prevent the oxidative damage caused by H_2_O_2_ toxicity with a potency of the protective effect displayed by FK (extracted using de-ionized water), which significantly (*p* < 0.01) increased CAT to 3.63 ± 0.61 U/mg pro. min, increased SOD to 29.98 ± 4.87 U/mg pro., and increased GSH content to 18.99 ± 0.75 μmol/mg pro. The ROS levels (Figure 4) of NFK and FK were 334.10 ± 14.53% and 312.97 ± 31.86%, respectively, which are significantly (*p* < 0.01) lower than the ROS level in the damage group. In PC12 cells, H_2_O_2_ induces overproduction of intracellular ROS and inhibition of ROS formation is protective against H_2_O_2_ cytotoxicity [35,36]. Flavonoid cloud decreased H_2_O_2_-induced ROS production and protected PC12 cells from cytotoxicity.

### 2.4. NFK and FK against Apoptosis of PC12 Cell as Revealed by Flow Cytometer Analysis

**Figure 5:**
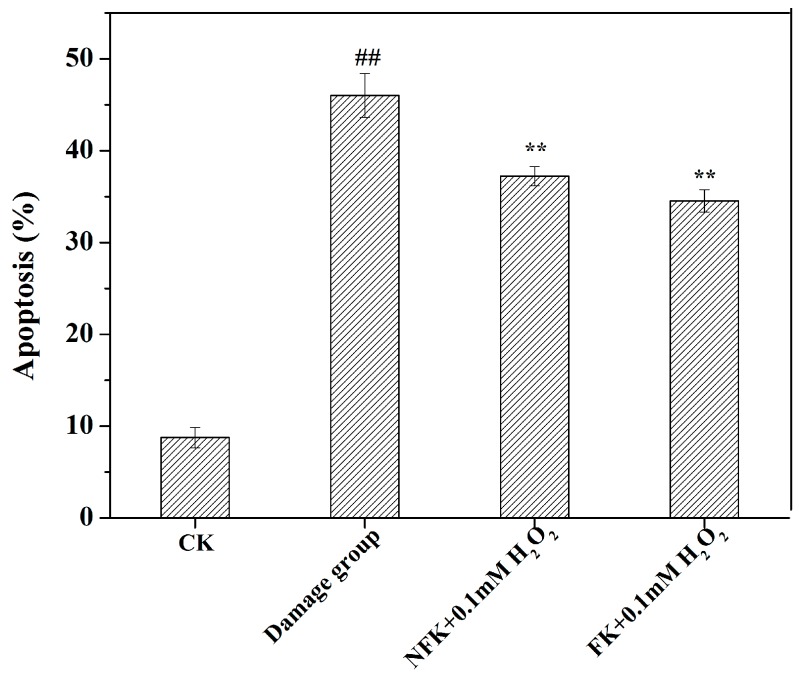
NFK (10 mg/mL) and FK (10 mg/mL) prevent H_2_O_2_-induced PC12 cell apoptosis. The probe Annexin V-FITC/PI was used to determine the cell apoptosis. Data are presented as mean ± S.D., (*n* = 3). ^##^ *p* < 0.01 compared with the control cells; ** *p* < 0.01 compared with the damage group.

**Figure 6:**
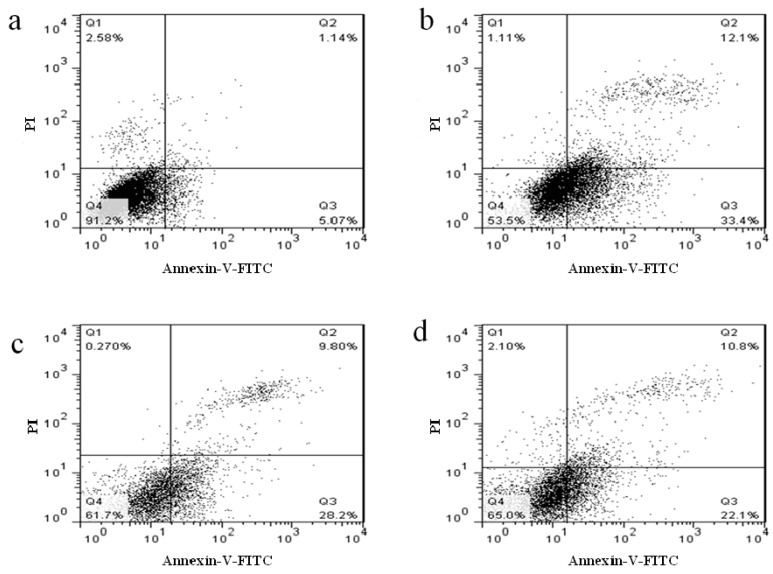
Cell apoptosis was measured by labeling the cells with Annexin-V-FITC and counterstaining with PI. (**a**) CK; (**b**) damage group; and (**c**,**d**) sample group. The numbers indicate the percentage of cells in each quadrant (lower left: FITC−/PI−, intact cells; lower right: FITC+/PI−, apoptotic cells; upper left: FITC−/PI+, necrotic cells; and upper right: FITC+/PI+, late apoptotic cells).

Based on the possible promotion effect of NFK and FK on proliferation of H_2_O_2_ damaged PC12 cell, NFK and FK possibly play some protective roles to these cells [37,38]. Apoptosis of PC12 cell induced by 0.1 mM H_2_O_2_ was investigated through flow cytometry. Compared with that of the untreated group (CK group), the apoptotic cell of PC12 cell exposed to 0.1 mM for 2.5 h significantly increased (*p* < 0.01), suggesting that the PC12 cell were damaged upon exposure to H_2_O_2_, and the apoptosis rate was 46.03 ± 2.41% (Figure 5). However, when the cells were pre-incubated with NFK and FK prior to exposure to 0.1 mM H_2_O_2_, the observed apoptosis rate was significantly attenuated (Figure 5 and Figure 6). When cells were pre-incubated for 0.5 h with NFK and FK extracted using deionized water before exposure to 0.1 mM H_2_O_2_, the cell viability was enhanced to 37.21 ± 1.05% and 34.54 ± 1.22%, respectively. Furthermore, we determined the cytotoxic effect of NFK and FK on PC12 cell, and the results revealed that NFK and FK effectively protected the PC12 cells against damage induced by 0.1 mM H_2_O_2_.

### 2.5. Cell Cycle Analysis via Flow Cytometry

**Figure 7:**
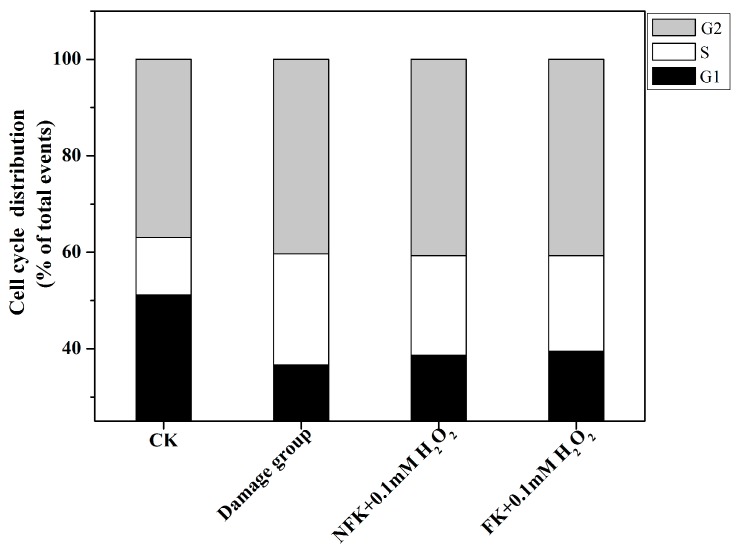
Effects of NFK (10 mg/mL) and FK (10 mg/mL) on H_2_O_2_ induced PC12 cell cycle as determined through flow cytometry.

**Figure 8:**
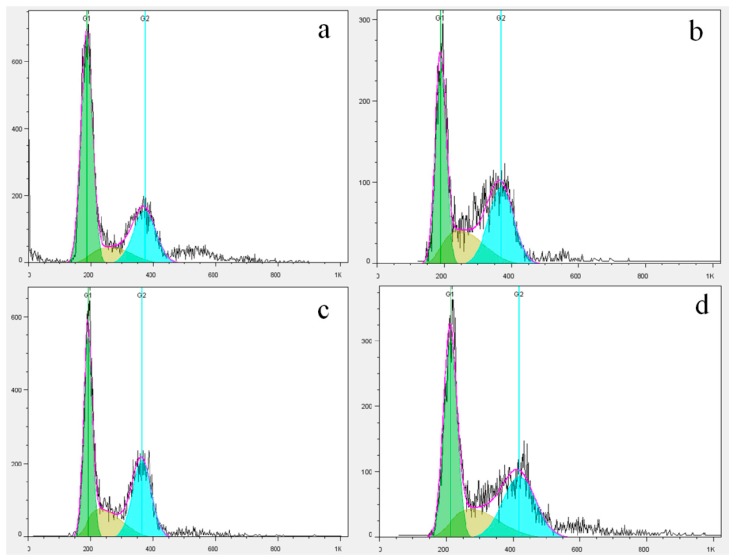
Cell cycle distribution of PC12 in (**a**) CK; (**b**) damage group and (**c**,**d**) sample group were analyzed through flow cytometry (■ G1, ■ G2, ■ S).

To analyze the effect of NFK and FK on PC12 cell, we determined the cell cycle distribution via flow cytometry which is evaluated using PI marking in the presence of samples extracted using de-ionized water extracted. Figure 7 and Figure 8 showed that the G1 phase of PC12 cell significantly decreased and the S phase significantly increased compared with those of the 0.1 mM H_2_O_2_ damage group. The PC12 cell were pretreated with NFK and FK for 0.5 h and then treated with 0.1 mM H_2_O_2_ for 2.5 h, the G1 phase significantly increased, whereas the S phase significantly decreased compared with those in the damage group. These results demonstrated that the NFK and FK could protect the PC12 cell against damage caused by 0.1 mM H_2_O_2_.

### 2.6. Determination of Intracellular Caspase-3 Activity

**Figure 9:**
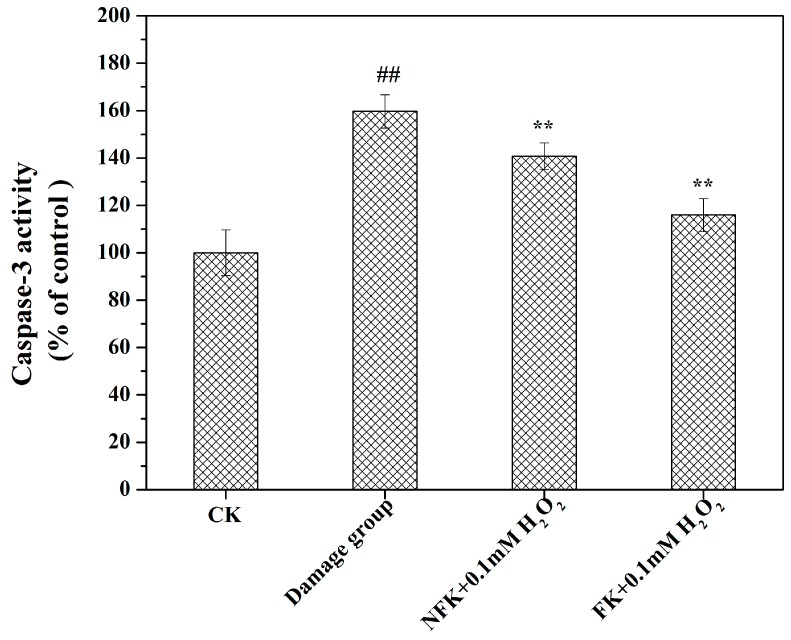
Effects of caspase-3 activity of NFK (10 mg/mL) and FK (10 mg/mL) on H_2_O_2_-treated PC12 cells. Data are presented as mean ± S.D., (*n* = 3). ^##^ *p* < 0.01 compared with the control cells; ** *p* < 0.01 compared with the H_2_O_2_ damage group.

Caspase-3 is a protease that is usually activated in response to cell death, and it catalyzes the breakdown of several vital cellular proteins [7]. To determine the pathway through which the NFK and FK could effectively protect the apoptosis of PC12 cell, we used a colorimetric detection assay kit used to determine caspase-3 activity. As shown in Figure 9, caspase-3 activity in CK of PC12 cell has lower significance compared with damage group. However, the 0.1 mM H_2_O_2_ induced caspase-3 activity was obviously attenuated in the sample group. Moreover, the NFK and FK extracted using deionized water could reduce caspase-3 activity by 140.75 ± 5.64% and 115.93 ± 6.92% of control respectively. By contrast, NFK reduced the caspase-3 activity at a lower extent compared with the FK. These results indicated that NFK and FK treatments could effectively block the damage induced by 0.1 mM H_2_O_2_ by increasing caspase-3 activity in PC12 cell. Inhibition of caspase-3 activation decreases the apoptosis of neurons. In the neuroprotection of pramipexole, caspase-3 activation induced by H_2_O_2_ is alleviated [39]. In the present work FK and NFK extracted by water inhibited caspase-3 activation induced by H_2_O_2_ which was involved in the protection for PC12 cells.

The present study results similar report by the Baohua et al. [16], that the d-β-hydroxybutyrate inhibited the apoptosis of PC12 cells induced by H_2_O_2_ via inhibiting oxidative stress. H_2_O_2_ can cause a series of oxidative stress through cells to induce the release of cytochrome C from the mitochondria, thus inducing cell apoptosis. Therefore, by measuring a series of antioxidant enzyme activities and antioxidant contents in PC12 cells, we can reflect the damage of PC12 cells induced by H_2_O_2_ and the protective effect of samples on cells. Apoptosis is a more complex process, which causes many causes and ways of cell apoptosis. The flow cytometry showed that flavonoid rich extract of FK and NFK could significantly reduce the apoptosis rate of PC12 cells H_2_O_2_ damage, further studies showed that FK and NFK can alleviate H_2_O_2_ caused S phase arrest phenomenon, reduce the activity and the key enzyme Caspase-3 in the apoptotic process to achieve.

## 3. Materials and Methods

### 3.1. Materials and Reagents

Dulbecco’s modified Eagle’s medium (DMEM) and fetal bovine serum (FBS) were purchased from Gibco BRL (Grand Island, NY, USA). H_2_O_2_, 3-(4,5-dimethylthiazol-2-yl)-2,5-diphenyltetrazolium bromide (MTT), and dimethyl sulfoxide (DMSO) were purchased from Shoude Biological Co., Ltd. (Nanjing, China). Puerarin, daidzin, glycitin, daidzein, glycitein, genistein and shikimic acid were purchased from Sigma-Aldrich Chemical Co. (St. Louis, MO, USA). Annexin V-FITC/PI apoptosis detection kit was purchased from rom Beyotime Institute of Biotechnology (Beijing, China). 2,7-Dichlorofluorescein dictate (DCFH-DA) ROS kits were purchased from Sigma-Aldrich. Lactate dehydrogenase (LDH), superoxide dismutase (SOD), catalase (CAT) and reduced glutathione (GSH) assay kits were procured from Nanjing Jiancheng Bioengineering Institute (Jiangsu, China). All other chemicals and reagents were of analytical grade. Rat pheochromocytoma line 12 (PC12) cell was purchased from the Institute of Biochemistry and Cell Biology (Shanghai, China).

### 3.2. Preparation of NFK and FK Extraction

Kudzu root (*Pueraria lobata*) was purchased from a medical supply company in Jiangsu, China. The samples were authenticated by reliable traditional Chinese herb experts. The fungus used to ferment the kudzu root (*Pueraria lobata*) was extracted from Fu brick dark tea in the laboratory. The kudzu root (*Pueraria lobata*) was sterilized in an SX-300 Autoclave (TOMY Seiko Co., Ltd., Tokyo, Japan) at 121 °C for 15 min. After cooling to room temperature, 100 g of the autoclaved kudzu root (*Pueraria lobata*) was inoculated with 3 mL of *E. cristatum* spore suspension and then incubated at 28 °C for 10 days (FK was fermented by *E. cristatum*). After fermentation, all samples were lyophilized and ground using an electric grinder (SF 180, Zhouxiang Phamaceutical Machinery Co., Ltd., Shanghai, China). Flour was passed through a 0.2 mm sieve and then stored in the dark at −20 °C for further analysis.

The NFK and FK were extracted using deionized water, the sample extracts were evaporated under a reduced pressure of 40 °C and then freeze drying. The sample weighing a certain quality was re-dissolved in deionized water, and the concentration of the extracts was 10 mg/mL and this concentration was used to the cell protective assay. The above samples were filtered using sterilized 0.2 µm filter membrane before being used in cell assay. The samples were analyzed by RP-HPLC using a Waters 2695 system (Aglient Technologies, Wilmington, DE, USA) with A-AORBAX SB-C 18 reverse-phase column, 4.6 × 200 mm, 5 µm particle size (Eclipse plus, Aglient, Technologies). The analysis empower software was used for controlling the instruments, for data acquisition and processing. The mobile phase consisted of deionized water as solvent A (contain 0.1% trifluoroacetic acid) and acetonitrile as solvent B and the solvent flow rate was 0.7 mL/min. The elution system was: 0 (95% B), 15 min (85% B), 32 min (75% B) and 45 min (50% B). An auto injector was used to inject 10 µL of the test solution into the RP-HPLC system. The wavelength used to monitor was set at 254 nm. The results were calculated by different standard curves and expressed as µg/g of dry weight sample.

### 3.3. Cell Culture and Treatment

PC12 cell were maintained in DMEM supplemented with 10% FBS, 100 U/mL penicillin, and 100 μg/mL streptomycin according to the method described by Pavlica et al. [38]. In all experiments, PC12 cell were seeded in 96-well (1.5 × 10^5^ cells/well) culture plates or 6-well culture plates (2.5 × 10^5^ cells/well) and then incubated at 37 °C in a humidified incubator containing 5% CO_2_ and 95% air for 24 h. The cells were pre-incubated for 0.5 h with NFK and FK extracted using deionized water, H_2_O_2_ (0.1 mM) was subsequently added into the medium and allowed to stand for 2.5 h.

### 3.4. Cell Survival Assay and LDH Activity

Cell viability was determined using MTT colorimetric assay as described by Fang et al. (2016) [40]. After treatment, the medium was removed, and the cells were incubated with 100 µL of 0.5 mg/mL MTT solution for 4 h at 37 °C. The medium was carefully removed, and dark blue formazan was dissolved with 150 µL of DMSO for 10 min at room temperature. Absorbance was measured at 570 nm by using a micro-plate reader. Cell viability was expressed as a percentage of control group, the cell viability of which was set as 100%. Cells were observed under an inverted microscope (ECLIPSE TE2000-S, Nikon, Tokyo, Japan).

Plasma membrane damage in PC12 cell was determined by the release of LDH into the medium [41]. After exposure of PC12 cell to H_2_O_2_ (0.1 mM) in the presence or absence of NFK and FK treatment for 2.5 h, the medium was collected to determine the LDH activity by using a commercially available assay kit according to the manufacturer’s protocol. The absorbance of the sample was measured at 450 nm. The LDH activity was expressed as U (reaction with the medium for 15 min at 37 °C producing 1 µM pyruvic acid) per liter.

### 3.5. Determination of SOD, GSH, CAT and ROS Levels

Following treatment of cells with NFK and FK, the medium was removed and the cells were washed twice with PBS. Cells were collected, centrifuged, re-suspended in PBS (0.5 mL), dissociated by cell lysis buffer, and centrifuged for 15 min (12,000 rpm, 15 min). The supernatant was used to measure the CAT and SOD activities 34 (Pavlica et al., 2010) and intracellular GSH contents by using an assay kit according to the manufacturer’s instruction [42]. SOD activity was expressed as U/mg protein, CAT activity was expressed as U/mL, and GSH content was expressed as µM/mg pro. Intracellular proteins were detected using the BCA methods and expressed as mg/mL.

Intracellular ROS production [29] was measured using the fluorescent probe DCFH-DA, which can cross cell membrane and can be subsequently hydrolyzed by intracellular esterase to non-fluorescent DCFH [43]. After NFK and FK treatment, cells on the 6-well plates were incubated with 10 µM DCFH-DA in DMEM and cultured for 0.5 h. The cells were harvested and then suspended in PBS. Fluorescence intensity was measured by a FACSCalibur (Becton, Dickinson and Company, Franklin Lakes, NJ, USA) flow cytometer at an excitation and emission wavelengths of 488 and 525 nm, respectively. Intracellular ROS was expressed as percentage of control.

### 3.6. Flow Cytometry Analysis of Apoptosis

Flow cytometry analysis was performed to identify and quantify the apoptotic cells by using an Annexin V-FITC/PI apoptosis detection kit. In brief, cells were treated with NFK and FK, harvested, washed twice with ice-cold PBS, re-suspended in binding buffer, and mixed with Annexin V-FITC and propidium iodide (PI). The cells were incubated in the dark for 0.5 h at room temperature [36], and then analyzed via flow cytometry (Becton, Dickinson and Company, Franklin Lakes, NJ, USA). Cell apoptosis was expressed as a percentage of control.

### 3.7. Cell Cycle Analysis

To elucidate the nuclear changes occurring during apoptosis, we harvested the NFK and FK treated cells were harvested through centrifugation and washed them twice with ice-cold PBS before fixation in 70% ethanol for 2 h or for more than 12 h at 4 °C. The cells were subsequently washed twice with PBS, and re-suspended in 50 µg/mL RNase at 37 °C for 0.5 h, and incubated with 25 µg/mL PI for 0.5 h at 4 °C in the dark. The cells were analyzed using a flow cytometer (Becton, Dickinson and Company, Franklin Lakes, NJ, USA), and all histograms were analyzed by Flowjo software, San Carlos, CA, USA.

### 3.8. Intracellular Caspaes-3 Activity Assay

Intracellular caspase-3 activity was determined using a caspase-3 activity detection assay kit. The PC12 cells were treated with NFK and FK, after cell lysis and centrifugation at 12,000 rpm for 15 min at 4 °C, the supernatant from lysed cells was added into the reaction mixture containing dithiothreitol and caspase-3 substrate (*N*-acetyl-Asp-Glu-Val-Asp *p*-nitroanilide) and then further incubated for 1 h at 37 °C. The absorbance of the chromophore *p*-nitroanilide was detected by a microplate reader at 405 nm [44]. Intracellular protein content was measured by using the Broadford method and expressed as mg/mL. PC12 cells were cultured as control group, and data were expressed as a percentage of control.

### 3.9. Statistical Analysis

Data were expressed as mean ± S.D. from at least three independent experiments. Student’s *t*-test was used to compare the means between control and damage group and between the sample group and damage group. Statistical analyses were performed using one-way ANOVA. *p*-Values of <0.05 indicated statistical significance.

## 4. Conclusions

Isoflavonoid compounds are the active compounds in kudzu root, and their amounts were increased by *E. cristatum* HC-18 fermentation, for example, the daidzein increased about 1.3 times, and there are reports reporting that microbe can increase the daidzein contents [45]. The NFK and FK were used to effectively protect PC12 cell by H_2_O_2_-induced damage. The results indicate that the NFK and FK extracted using de-ionized water, and they protected the cell membrane, increased the intracellular antioxidase system load, and inhibited H_2_O_2_-induced apoptosis by scavenging ROS, and regulating the cell cycle. Moreover, this study demonstrated that the fermented kudzu can be developed to prevent and cure oxidation-related diseases, and that it presents potential applications in medical health care.

## Figures and Tables

**Table 1:** Special isoflavonoid compounds of NFK and FK. The results are expressed as µg/g. DW.

Special Compounds	NFK	FK
One: Puerarin	1903.56 ± 62.88 A	2395.26 ± 78.65 B
Two: Daidzin	147.13 ± 3.39 A	69.82 ± 4.22 B
Three: Glycitin	129.04 ± 1.62 A	91.45 ± 9.40 B
Four: Genistin	16.02 ± 0.35 A	11.42 ± 0.60 B
Five: Shikimic acid	141.18 ± 3.82 A	149.55 ± 10.21 B
Six: Daidzein	145.62 ± 23.91 A	188.77 ± 12.47 B
Seven: Glycitein	43.87 ± 1.79 A	46.40 ± 2.80 B
Eight: Genistein	2.16 ± 0.25 A	2.78 ± 0.20 B

Each value represents the mean ± S.D. (*n* = 3). Means being different capital letters (A,B) within a row under the same fermentation condition showed a significant difference (*p* < 0.05) between NFK and FK.

**Table 2:** Effect of NFK and FK on LDH contents, CAT and SOD activities, and GSH contents in PC12 cells.

LDH, CAT, SOD Activities, GSH Content	Control Group	Damage Group	Sample Group	
			NFK	FK
LDH (U/L)	292.05 ± 53.42	566.45 ± 79.43 ^##^	468.45 ± 19.20 a **	443.80 ± 33.26 b **
CAT (U/mg pro.)	9.25 ± 1.48	1.61 ± 0.30 ^##^	2.67 ± 0.27 a **	3.63 ± 0.61 b **
SOD (U/mg pro.)	116.26 ± 5.82	21.07 ± 2.81 ^##^	27.88 ± 1.716 a **	29.98 ± 4.87 a **
GSH (mmol/mg pro.)	29.13 ± 5.31	6.17 ± 0.39 ^##^	14.28 ± 0.54 a **	18.99 ± 0.75 b **

Each value represents mean ± S.D. (*n* = 3). Means being different small letters (a,b) indicate a significant difference (*p* < 0.05) between the same concentrations of NFK and FK. ^##^ *p* < 0.01 indicates significant difference between the control group and the damage group, ** *p* < 0.01 indicates significant difference between sample group and damage group, pro. represents protein.
